# Dye-doped spheres with plasmonic semi-shells: Lasing modes and scattering at realistic gain levels

**DOI:** 10.3762/bjnano.4.110

**Published:** 2013-12-30

**Authors:** Nikita Arnold, Boyang Ding, Calin Hrelescu, Thomas A Klar

**Affiliations:** 1 Institute of Applied Physics, Johannes Kepler University, 4040 Linz, Austria

**Keywords:** gain, metamaterials, nanophotonics, plasmonics, spaser

## Abstract

We numerically simulate the compensation of absorption, the near-field enhancement as well as the differential far-field scattering cross section for dye-doped polystyrene spheres (radius 195 nm), which are half-covered by a silver layer of 10–40 nm thickness. Such silver capped spheres are interesting candidates for nanoplasmonic lasers, so-called spasers. We find that spasing requires gain levels less than 3.7 times higher than those in commercially available dye-doped spheres. However, commercially available concentrations are already apt to achieve negative absorption, and to narrow and enhance scattering by higher order modes. Narrowing of the plasmonic modes by gain also makes visible higher order modes, which are normally obscured by the broad spectral features of the lower order modes. We further show that the angular distribution of the far-field scattering of the spasing modes is by no means dipole-like and is very sensitive to the geometry of the structure.

## Introduction

Noble metal nanoparticles are of current interest both in fundamental and applied science because of their localized plasmonic resonances (LPR) in the visible and near infrared range of the optical spectrum. The simplest nanoparticle geometry is spherical, but the spectral position of the nanoparticle plasmon of a solid sphere can only be tuned by increasing its radius (given a specific refractive index of the embedding medium), which leads to a redshift with increasing radius [1]. The price one has to pay for this size-based wavelength-tuning is, however, a substantial spectral broadening due to radiative damping. An alternative way to tune the LPR spectrally is to change the shape of the nanoparticle. First, one can relax the radial homogeneity of the nanoparticle and turn from solid nanoparticles to noble metal nanoshells [2–3]. Second, one can also relax the angular symmetry and turn from nanoshells with spherical symmetry to semi-shells, sometimes also called nano-caps or nano-cups. Such semi-shells can be produced either via the evaporation of noble metals on top of dielectric spheres [4–6], via the electrochemical deposition through a self-assembled template of dielectric spheres [7], via the attachment of seed particles to dielectric spheres that are partially embedded in a polymer matrix and a subsequent electroless plating [8], or via opening holes in originally closed shells via e-beam sputtering [9] or ion beam milling [10].

The semi-shells show a rich spectrum of localized plasmon resonance modes that was investigated analytically and numerically [11–13]. It became common to label the different modes according to the symmetries of the modes of the spherically symmetric closed metallic shells. The semi-shell modes can be derived from the closed-shell modes by slowly opening the closed shell. Labeling is hence carried out by using the multipolar and azimuthal numbers (*l,m*) [7,11]. While in a spherically symmetric shell, each multipolar mode is (2*l*+1) times degenerate, the symmetry breaking that is introduced by opening a hole in the shell not only lifts this degeneracy, but also introduces a further mode splitting via the coupling of the shell plasmons with the rim plasmon modes of the circular opening [11]. The excitability of the localized plasmon modes depends on the polar and azimuthal angles of the direction of illumination and on the orientation of the electric field vector. In a recent experimental work, some of the most prominent eigenmodes of metallic voids have been imaged using a near-field microscope [14]. A further prominent feature of semi-shells with broken symmetry (compared to angularly symmetric shells) is their capability to scatter light preferentially into certain directions [15–16]. Recently, we have shown that localized plasmonic modes of the semi-shells are apt to direct the fluorescence from the dyes contained in their dielectric cores into the forward direction [17]. Plasmonic semi-shells or nanovoids have also been used for important applications such as biosensing [18], plasmon-enhanced solar cells [19–20], or as substrates for surface-enhanced Raman scattering [21–22] and coherent anti-Stokes Raman scattering [23].

A severe problem for all plasmonic applications is the damping of plasmons due to Ohmic losses in the metal and due to radiative losses. However, a solution for this dilemma is possible because plasmons are Bosons and can hence be emitted via stimulated emission [24]. This might be used to minimize losses in metamaterials [25–26] and it has also been proposed for the compensation of losses in plasmonic shells [27–28]. Loss-compensation in the case of solid gold nanospheres embedded in a gain medium has been experimentally verified by Noginov et al. [29] and more recently confirmed by Strangi et al. [30]. The cancellation of the losses in hybrid materials that comprise resonant nanoparticle plasmons and gain materials finally leads to a self-sustaining laser-like generator called the spaser [31–36]. In this contribution, we find that there is a finite range of gain values in which the absorption is overcompensated within a certain wavelength region, but the spaser does not self-start yet. The existence of such a finite range has been debated before [37–41].

A true nano-spaser based on localized plasmons should be sub-wavelength confined in all three dimensions (3D). Experimentalists approached such 3D nano-spasers within the last few years [42–48]. The smallest nano-spaser so far has been claimed by a team around Noginov, Shalaev and Wiesner [49] who reported that gold nanoparticles with a diameter of 14 nm, covered by a silica shell of 15 nm thickness, doped with the dye OG-488 show laser emission. The nanostructures reported in this experimental study were, however, fully spherically symmetric. Very recently, it has been proposed that symmetry breaking might have advantages because a low gain threshold is required [50] and the coherent emission becomes directional [51]. In both theoretical papers, a geometry was assumed, in which a dye-doped dielectric sphere was covered by a semi-shell of noble metals.

The authors of [50–51] assumed metal-capped and dye-doped spheres comprising a spectrally flat, dispersion-less gain. This does not reflect realistic dye molecules, which provide gain only within a limited spectral range. However, it is a useful approach to search for the spectral mode that will lase most easily. In this work, we would like to take the complementary approach by starting with two given Lorentzian lines for the molecular absorption and the Stokes-shifted emission of a given realistic dye molecule that fluoresces in the visible region. We tune specific plasmonic resonances into the emission maximum of the dye molecules by adjusting the thickness of the silver caps on the dye-doped spheres. In particular, we assume a doping concentration, a size of the spheres and the type of dye molecules as given by the commercially available polystyrene spheres “Firefli* Fluorescent Green” from Thermo-Scientific, Waltham, MA, USA, which we have used in an experimental study on the spectral and directional Purcell effect [17]. With this self-restriction to the parameters of commercially available dye-doped spheres, we make a realistic estimate of how far away one is from nanoparticle spasing (we find: a factor of 3.7 in necessary gain, only) and which other effects, such as a spectral narrowing of the scattering cross section or a change in absorption should already be observable in the case of the available gain. We find that narrowing of the Mie scattering spectrum and small signal gain could become detectable.

## Numerical modeling

**Figure 1:**
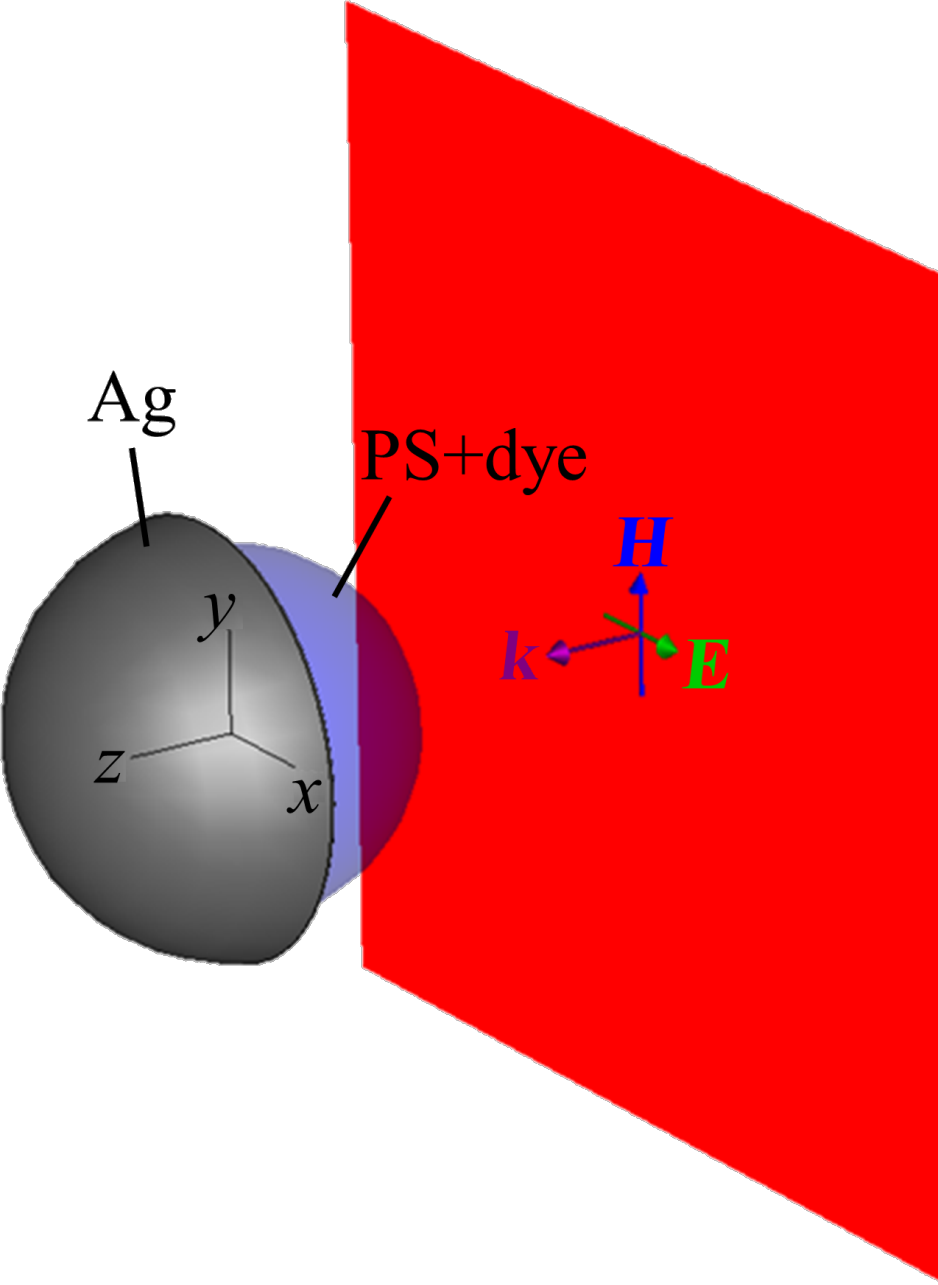
Geometry of the sphere and the illuminating plane wave. The ***k***-vector of the plane wave is defined by (θ,φ), in which θ denotes the angle between the ***k***-vector and z axis and φ is the polar angle counted from the x-axis. The shown geometry hence depicts the (0,0) illumination.

Figure 1 depicts the geometry of the silver capped spheres and the orientation of the incoming electromagnetic wave. A dye-doped polystyrene (PS) sphere of 390 nm diameter is capped with a semi-shell of silver. The coordinate system is fixed such that the z-axis defines the axis of rotational symmetry of the semi-shell and points inwards into the metallic cap. The direction of the ***k***-vector of the linearly polarized plane wave is given by two spherical angles (θ,φ) in which θ denotes the polar angle between the ***k***-vector and the z axis, and φ denotes the azimuthal angle in the x–y plane. The electric field always remains in the x–z plane. In particular, the (0,0) direction denotes an illumination from the open, uncovered side of the PS sphere (as shown in Figure 1), while (180,0) denotes an illumination from the silver-capped side and (90,0) denotes an illumination from the side, in which the electric field is parallel to the z-axis such that axial plasmonic modes can be excited. The thickness of the silver shell and the concentration of the dye molecules are varied throughout the paper.

Realistic modeling of 3D plasmonic structures with gain in the visible-light range is non-trivial and requires certain care. As mentioned before, semi-shell structures possess many multipolar eigenmodes that are located closely together in frequency space [11], and many of the modes are easily overlooked when modeling is carried out without gain, because they are strongly damped because of the dispersion of the metal. If, however, damping is compensated by gain, the modes might become ultra-sharp and can still be overlooked if they are narrower than the frequency step used in simulations. The situation is somewhat similar to the scattering by weakly dissipating plasmonic spheres, in which very narrow higher order resonances can dominate [52]. These effects are even more pronounced for the anisotropic dielectric permittivity with transverse or longitudinal gain [53]. Computational details are discussed in the section “Numerical” at the end of the paper.

The dielectric constant of silver was taken from Johnson and Christy [54]. The dielectric constant of the gain material was described by a double Lorentzian function:

[1]
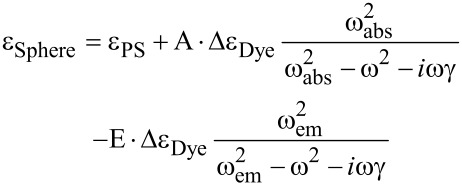


The coefficient Δε_Dye_ describes the strength of the transition (for the time being the same strength of absorption and emission is assumed [55]), ω_abs_ = 4.02·10^15^ s^−1^ (468 nm) and ω_em_ = 3.67·10^15^ s^−1^ (513 nm), γ = 5.0·10^14^ s^−1^ (Δλ_FWHM_ ≈ 60 nm), and we assume a purely real ε_PS_ = 2.6 for polystyrene. The coefficient A is set to “1” and the coefficient E is set to “0” if we assume a purely absorbing medium (all dye molecules in the ground state) and A = 0, E = 1 in case of a fully inverted medium. Coefficients between 0 and 1 are used to simulate partial inversions, in which A + E = 1 holds.

Unsaturated Lorentzian gain does not allow for simulations very close to the lasing threshold (leading to unphysical infinite scattering cross sections and a diverging gain [36,56]), nor does it have much of a physical meaning beyond the threshold in CW operation. However, a classical electromagnetic calculation with a Lorentzian gain is fully legitimate as long as the lasing threshold is approached from below [36]. Here, we define the extinction cross section as σ_ex_ = σ_sc_ + σ_ab_, irrespectively of the sign of σ_ab_.

To get realistic simulation parameters, we determined the chromophore density in dye-doped polystyrene (PS) spheres, which are commercially available from Thermo-Scientific (Waltham, MA, USA). Those spheres are doped with “Firefli* Fluorescent Green” dye with an absorption peak near 468 nm and an emission peak at 513 nm. The concentration of the dye molecules inside the PS spheres was derived by taking the optical density (OD) of a solution of dye-doped spheres and subtracting the scattering component of the OD. The concentration of the spheres was quantified by measuring the scattering strength at a wavelength far from the absorption of the dyes. From this, we estimated an absorption constant in the range of 2300 cm^−1^, which translates into a transition strength of Δε_Dye_ = 0.004. In the simulations, we often considered cases of pure absorption or pure emission, which correspond to the cases in which all the dye molecules are in the absorbing ground state or all dye molecules are in the emitting state respectively (full inversion, which is achievable in a four level system). We also considered a 50/50 combination of two Lorentzian lines, which corresponds to half of the chromophores being excited (A = E = 1/2 in Equation 1). For the dye concentration of these commercially available PS spheres, we do not observe any spasing, yet. Hence, we also considered substantially higher dye concentrations, i.e., we applied Δε_Dye_ > 0.004. Primarily for didactical reasons we also went to dye concentrations beyond the lasing threshold. As a reference, the peak of the imaginary part of the dielectric constant 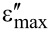 is related to Δε_Dye_ at the central emission wavelength via the equation 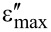 = Δε_Dye_·ω_em_/γ = 7.34 Δε_Dye_.

## Results and Discussion

### Matching the plasmonic resonance to the gain spectrum

**Figure 2:**
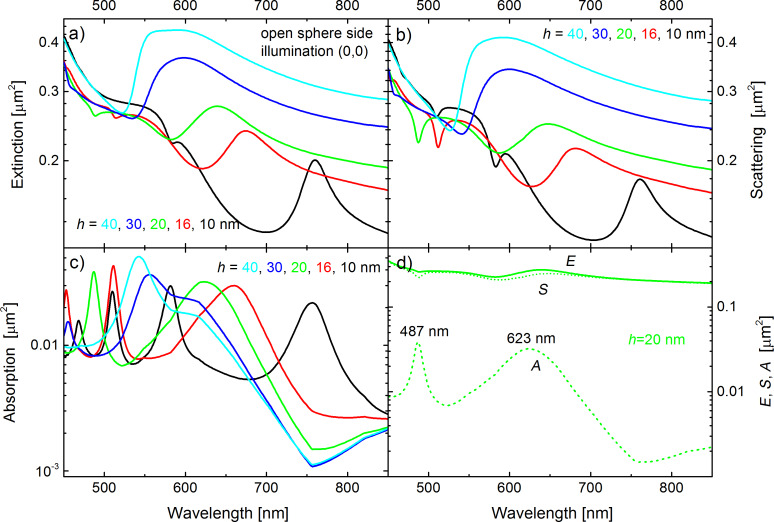
Cross sections of the (a) extinction, (b) scattering and (c) absorption of a PS sphere of diameter *d* = 390 nm, without fluorophores, and capped with a Ag semi-shell of variable thickness *h*, as given by the color coding. The incident plane wave was oriented in (0,0) direction, i.e., impinging from the open sphere side. The plasmonic resonances are most prominent in the absorption spectra. (d) compares the extinction (*E*), scattering (*S*) and absorption (*A*) spectra for the specific shell thickness *h* = 20 nm.

Before we include the gain material in the calculations, we tune the plasmonic resonance of the semi-shells to overlap with the emission band of the fluorophores by adjusting the thickness *h* of the silver caps on the undoped PS spheres of 390 nm diameter. Figure 2 shows the extinction, scattering, and absorption spectra in panels a,b,c, respectively, for thicknesses varying from *h* = 10 to 40 nm as indicated by the color coding. The linearly polarized, electromagnetic plane wave impinges the structure from the “open sphere side”, i.e., in the (0,0) direction as depicted in Figure 1. In Figure 2d all three cross sections are compared for the specific sample with *h* = 20 nm, showing that the total extinction spectrum is dominated by the scattering. However, the most prominent resonances are seen in the absorption spectra. On the black curve (*h* = 10 nm) in Figure 2c, one clearly sees several plasmonic maxima, for which the maxima become sharper with decreasing wavelength. When the thickness of the semi-shells is decreased, the series of absorption peaks shifts to the red. For instance, the peak near 510 nm corresponds to the third resonance (counted from the long wavelength side) in the case of the 10 nm thick semi-shell (black curve), but to the second resonance in the case of 16 nm thickness (red curve). At a closer look, one can observe some minor peaks, sometimes masked as a shoulder of a main peak. The spectrum of resonances is quite rich and it is not straightforward to assign a specific (*l,m*) mode to the peaks, which will become clearer later, when we use gain to sharpen the resonances. Only the longest-wavelength mode can safely be assigned to an *l* = 1 mode.

**Figure 3:**
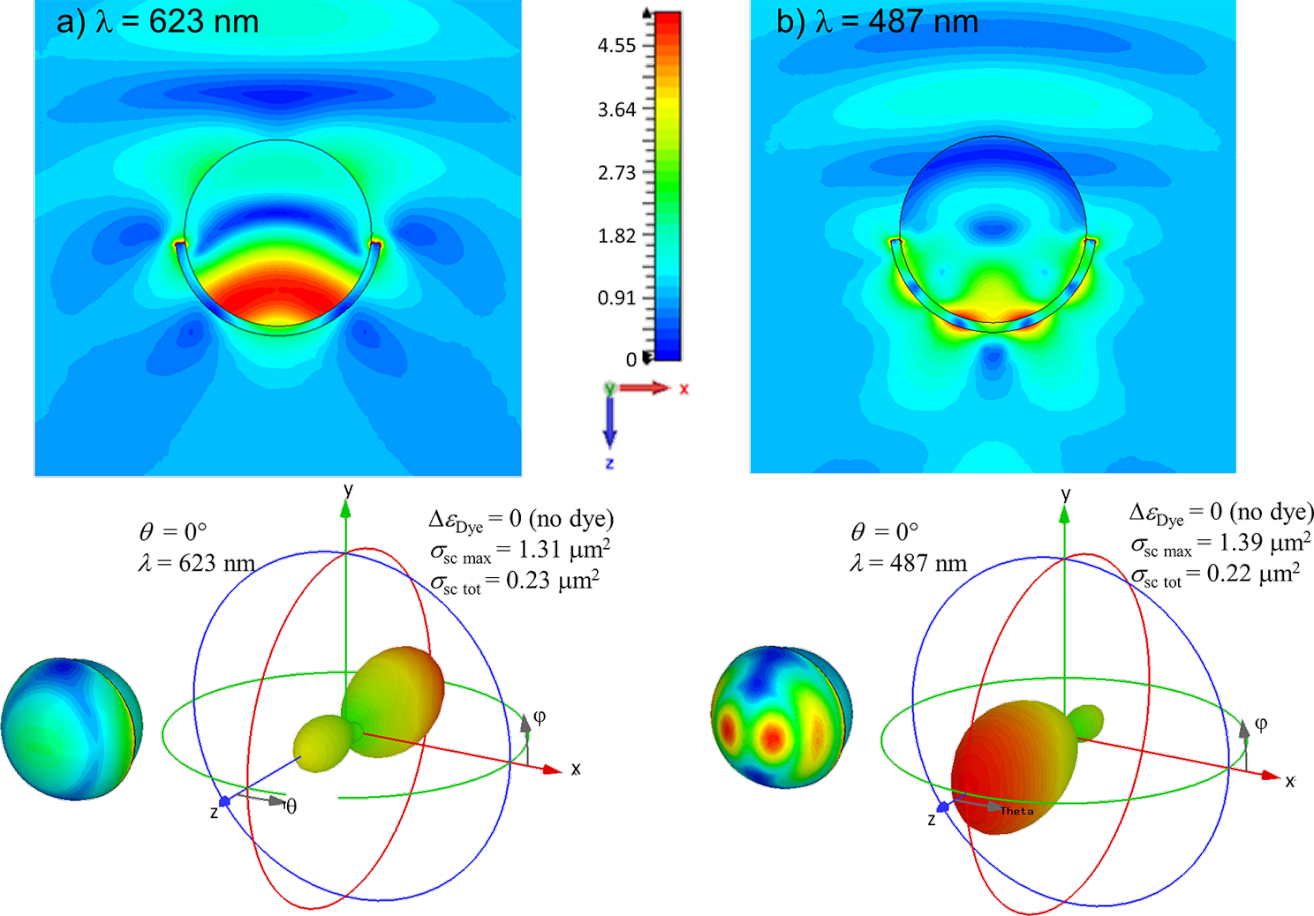
Mode characteristics of the two absorption maxima in Figure 2d ((0,0), i.e., sphere side illumination, *h* = 20 nm), at λ = 623 (a) and 487 nm (b). The upper images show the field enhancement |***E***|/*E*_0_ in the propagation–polarization x*–*z plane. The 3D graphs on the lower left of (a) and (b) show |***E***|/*E*_0_ in air near the surface of the semi-shell, i.e., the near-field just outside the structure. The mode at 623 nm is a “void like” dipolar mode (*l* = 1), while the mode at 487 nm is a *l* = 2 mode. The 3D spherical plots show the differential scattering cross sections on a linear scale. The maximum of the differential and the total σ_sc_ are given in the legends.

Figure 3 shows the mode profiles in case of the semi-shell with *h* = 20 nm. Specifically, it shows an *l* = 1 and an *l* = 2 mode with resonances in the absorption spectra at 623 and 487 nm, respectively (c.f. Figure 2d). The upper panels in Figure 3 show the field enhancement |***E***|/*E*_0_, in which *E*_0_ is the amplitude of the electric field in the incident plane wave. The images are cut in the x–z plane, i.e., the plane spanned by the ***k***-vector (z-axis) and the polarization of the electric field (x-axis). A three dimensional representation of the near field enhancement just outside the semi-shell structure is given on the lower left of both panels. At 623 nm, the plasmonic mode of the semi-shell can be derived from a pure dipolar resonance if the shell was not a semi-shell but a fully closed shell. Hence it is termed an *l* = 1 resonance [11]. Because of the opening of the semi-shell (with respect to the closed shell), the *l* = 1 resonances loose degeneracy and the mode depicted in Figure 3a is the transverse *l* = 1 mode (the axial mode is not excitable by a plane wave in the (0,0) direction) [16]. The mode at 487 nm is essentially an *l* = 2 mode. Both modes are constructively coupled to the rim mode [11] and originate from the long-wavelength *l* modes of the full shell [57]. The lower right graphs are polar graphs of the differential scattering cross section.

**Figure 4:**
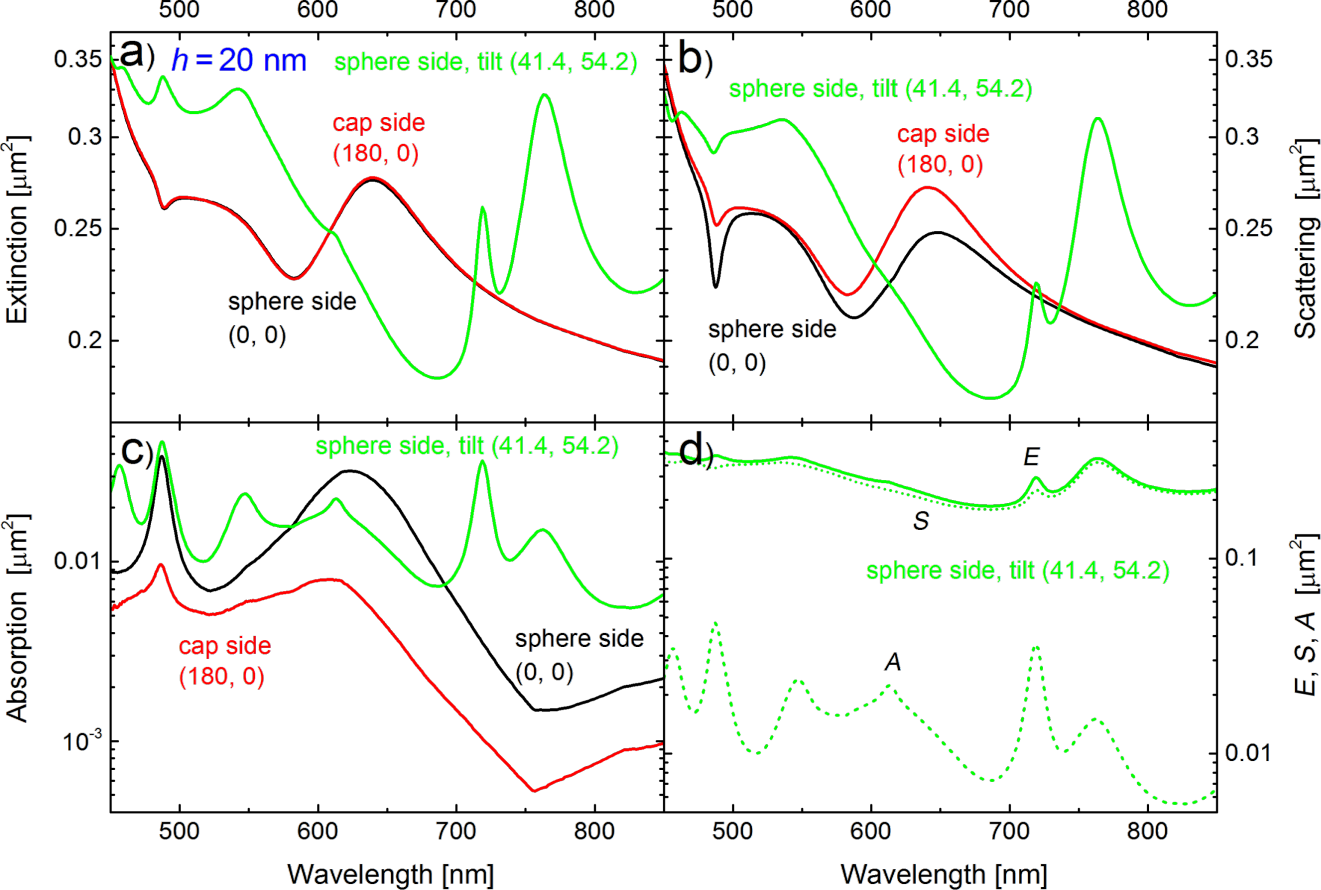
Dependence of the cross sections of the semi-shell structure with a core diameter *d* = 390 nm and a Ag thickness *h* = 20 nm on the direction of irradiation. Illumination from the open sphere side (0,0) (black curve), or from the Ag-capped side (180,0) (red curve), or from the sphere side with the plane wave impinging under the oblique angels θ = 41.4°, φ = 54.2° with the electric field in the x–z plane (green curve). Other parameters and notations are the same as in Figure 1. Illumination under oblique angles leads to many more resonances because of the loss of degeneracy by cylindrical symmetry.

Figure 4 shows the extinction, scattering, and absorption cross sections of the *h* = 20 nm semi-shell structure for different illumination directions. The cross sections for the (0,0) direction (illumination from the open sphere side) are redrawn from Figure 2d for comparison (black curves). Red curves correspond to illumination from the silver capped side, along the negative z-axis (180,0). Further, we compare both results with the cross sections obtained by an illumination under the oblique angles (41.4, 54.2). These angles sound a bit arbitrary, but they are equivalent to two consecutive rotations of the structure by −30° about the x- and y- axes, while leaving the incident wave intact. We see that the extinction cross sections of the sphere side (0,0) and the cap side (180,0) coincide, while the absorption and scattering cross sections differ in magnitude but not in the spectral shape. In contrast, there is a significant difference in the spectral shape for the (41.4, 54.2) orientation. Two new resonances appear (between 700 and 800 nm), the main resonance at 640 nm disappears in the scattering spectrum (while still being visible at 623 nm in the absorption). A clear absorption peak appears at 545 nm where there was only a weak shoulder under (0,0) or (180, 0) illumination, however, the *l* = 2 absorption peak at 487 nm is similar to what we observed for the (0,0) direction. This is an important finding, because we will spectrally overlap this *l* = 2 absorption peak with the emission line of the dye molecules in the subsequent sections of the paper. Figure 4d compares the extinction, scattering, and absorption cross sections for the case of the oblique incidence.

### Plasmons supported by gain

**Figure 5:**
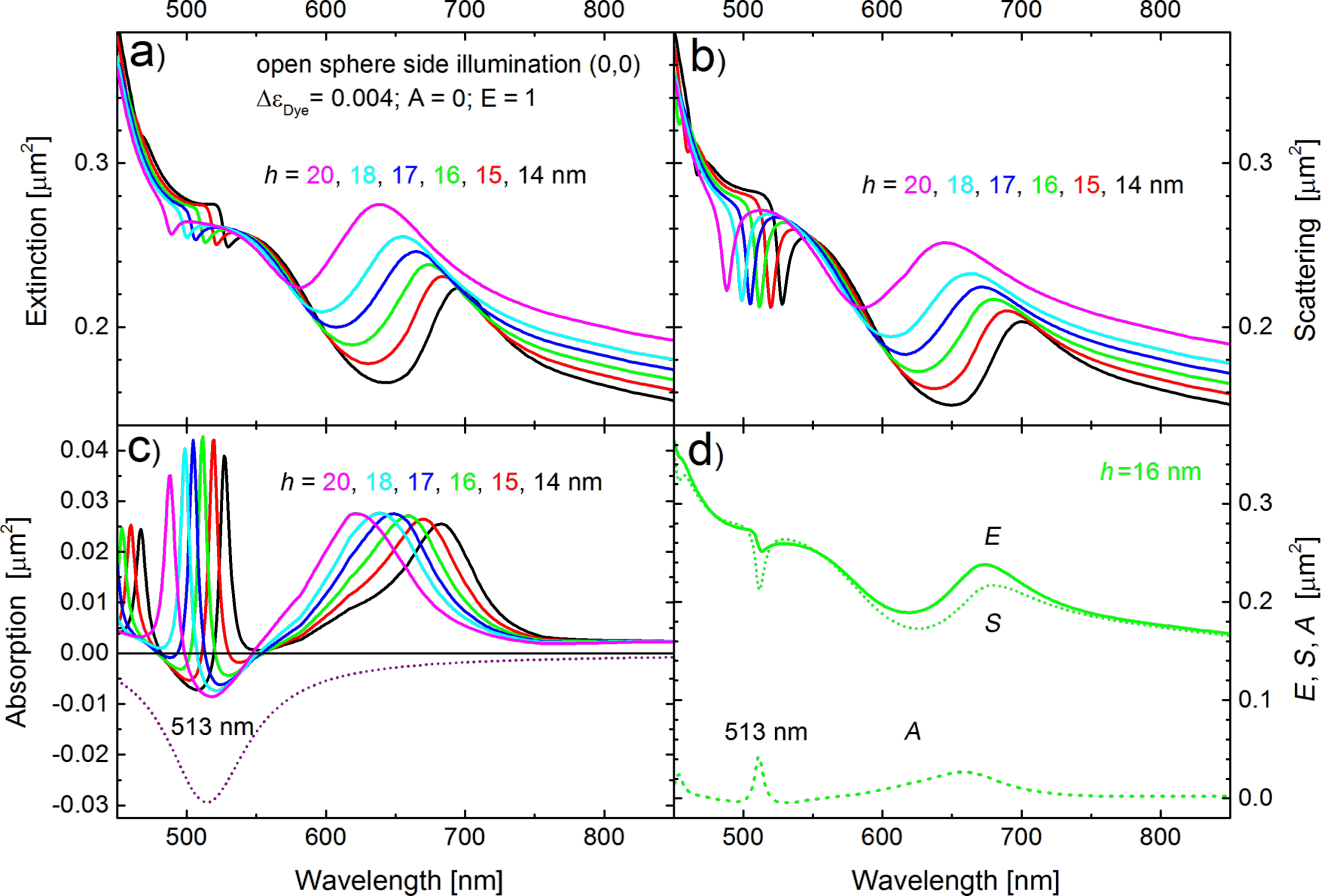
Fine-tuning of the Ag semi-shell resonances to the emission band of the dye. Parameters and the organization of panels a–d are the same as in Figure 2 apart from the thickness *h*, color coded in the plots, and ε_Sphere_ from Equation 1, which now includes dye molecules. Dimensionless values of Im(ε_Sphere_) are shown in panel (c) by a dotted curve.

In order to fine-tune the *l* = 2 absorption peak to the emission line of the dye molecules, we plot the corresponding negative Im(ε_Sphere_) into Figure 5c together with the absorption peaks of the semi-shells with thicknesses between 14 and 20 nm. As the shell thickness varies, the *l* = 2 absorption peak scans across the dye emission line. Figure 5a and Figure 5b show the corresponding extinction and the scattering cross sections and Figure 5d shows all three cross sections for the case of *h* = 16 nm. For all calculations shown in Figure 5 we used a dielectric constant ε_sphere_ defined in Equation 1, which includes the contribution of the dye molecules to the dielectric constant. The dotted line in Figure 5c represents Im(ε_Sphere_). We used Δε_Dye_ = 0.004 and ω_em_ matching the center of the emission line at λ = 513 nm. We further used A = 0 and E = 1 in Equation 1, which correspond to a full inversion of all dye molecules inside the PS spheres. This results in a negative absorption of our composite structure over a considerable spectral range. When *h* = 16 nm (green curves in Figure 5a–c) the *l* = 2 absorption resonance perfectly coincides with the center of the emission line at 513 nm. Besides, the absorption peak is the largest and the scattering is the lowest for this specific shell thickness as compared to the other thicknesses. We note in passing that the absorption cross sections in Figure 5c become negative for some wavelengths around the *l* = 2 peak at 513 nm and for Δε_Dye_ = 0.004. We will discuss this in more detail at the end of this paper. Figure 5d shows the extinction (*E*, solid curve), scattering (*S*, dotted) and absorption (*A*, dashed) cross sections for the semi-shell thickness *h* = 16 nm.

**Figure 6:**
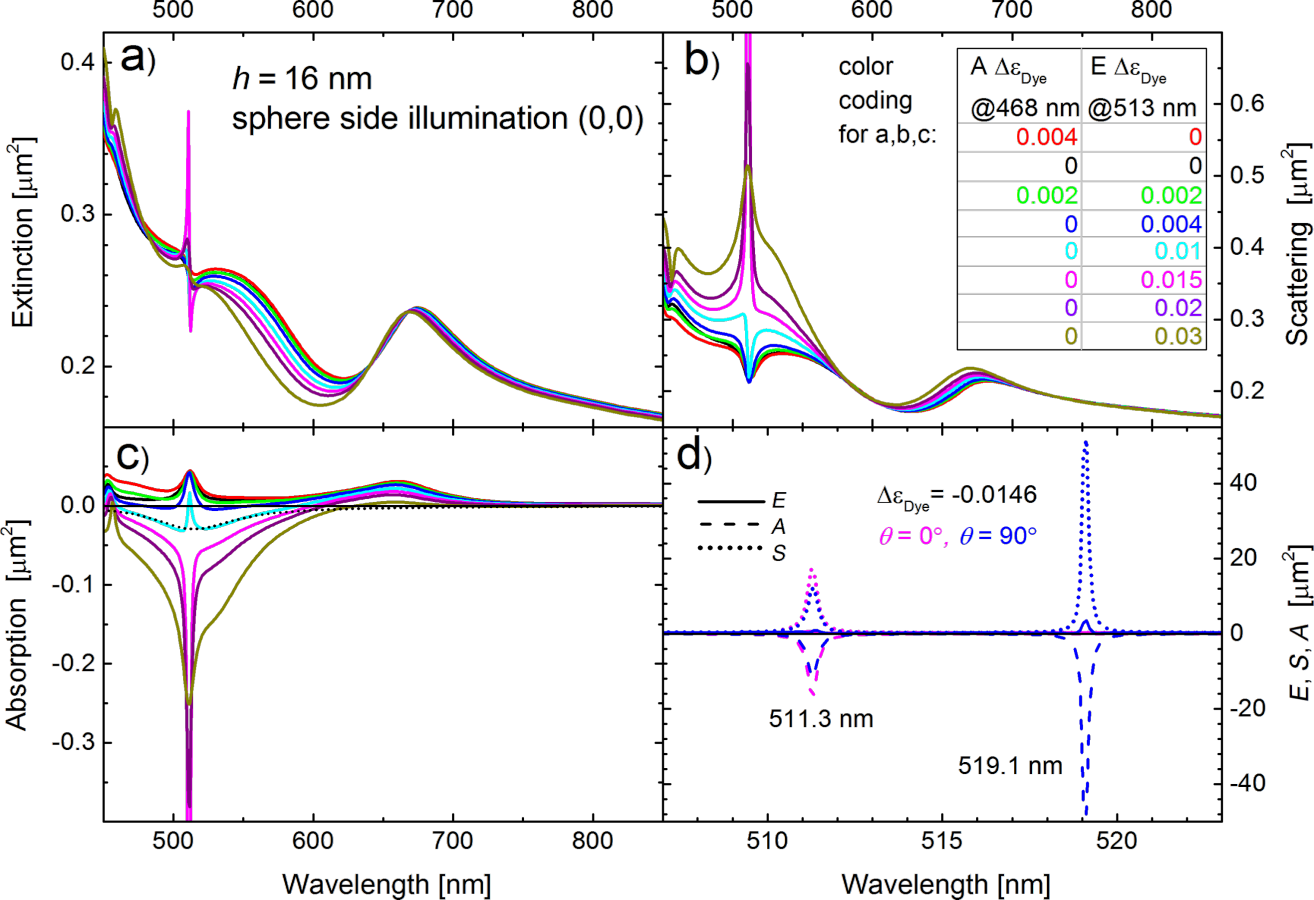
Approach to lasing for a semi-shell thickness of *h* = 16 nm. Parameters and order of panels a,b,c are the same as in Figure 2. The strengths of the absorption and the gain (cf. Equation 1) are color-coded as shown in the inset of (b). The dimensionless Im(ε_Sphere_) is shown in (c) by the dotted curve. Panel (d) shows a zoom of the extinction (solid curves), scattering (dotted) and absorption (dashed) spectra near the lasing threshold (Δε_Dye_ = 0.0146, A = 0, E = 1) close to the *l* = 2 resonance. The magenta lines refer to the sphere side irradiation (0,0), while the blue lines are calculated for an illumination from the x-direction (θ = 90°, φ = 0°) so that the incident light is polarized in axial z-direction. In this case a second resonance appears near 519 nm, in addition to the resonance near 511 nm, which exists for both orientations.

We now vary the amount of the active dye material inside the PS spheres in the simulations. Figure 6a–c shows the extinction, scattering, and absorption cross sections, respectively, for the case of a semi-shell thickness *h* = 16 nm, for which the emission line of the dye molecules and the absorption resonance of the semis-shell structures coincide. The strengths Δε_Dye_ of the absorption (a Lorentzian curve peaked at 468 nm) and the gain (a Lorentzian curve peaked at 513 nm) are color-coded as shown in the inset of Figure 6b. Hence, the cross sections of the undoped semi-shell structures are given by the black curve (Δε_Dye_ = 0), the case of a purely absorbing commercially available PS sphere (Δε_Dye_ = 0.004, A =1, E = 0 in Equation 1) is given by the red line, the fully inverted case is given by the dark blue curve and the 50% inverted case by the green curve. The cyan, magenta, purple, and olive curves correspond to PS spheres that are hypothetically doped with fully inverted dye molecules to yield E Δε_Dye_ = 0.010, 0.015, 0.020, and 0.030, respectively. Im(ε_Sphere_) is plotted into Figure 6c as a dotted curve for Δε_Dye_ = 0.004, A = 0 and E = 1.

The onset of lasing is given by singularities in the absorption and scattering cross sections and occurs near Δε_Dye_ = 0.0146, which means at concentrations less than 3.7 times larger than available in commercial PS spheres. Hence, we conclude that lasing should be possible in custom made PS spheres with a moderately enhanced dye concentration. No lasing is observed yet at the commercially available gain strength of Δε_Dye_ = 0.004, but the absorption cross section becomes slightly negative in the vicinity of the absorption peak (discussed in more detail below). When approaching laser threshold, the full width at half maximum (FWHM) of the scattering and absorption peaks goes to zero [51], which is an artifact of the purely classical calculation. Nevertheless, this singularity in cross sections indicates the transition from an amplifier to self-sustained lasing. A CW operation of a nano-spaser above the threshold (Δε_Dye_ > 0.0146) is possible only with continuous pumping, in which the field strength and linewidth are dictated by the gain saturation and spontaneous emission noise [36,56,58]. We do not discuss the thermal stability of such a system, which is strongly material-dependent.

In panels a–c of Figure 6, only the (0,0) illumination direction (open sphere side) is considered. Figure 6d compares the (0,0) direction (magenta curves) with the (90,0) direction (blue curves), in which the electric field is now polarized along the z-axis and hence allows for the excitation of axial cap plasmons. Note that the abscissa is zoomed in to span 507 to 523 nm, and the ordinate is enlarged to span −50 to 55 µm^2^ in order to show the almost diverging peaks for Δε_Dye_ = 0.0146. The solid lines in Figure 6d are the extinction cross sections, the dashed lines the absorption and the dotted lines the scattering cross sections. One clearly sees that the resonance at 511.3 nm is strongly excited in both cases, for (0,0) and for (90,0) illumination. However, a second resonance at 519.1 nm appears for the (90,0) illumination, which is not excitable with (0,0) illumination for symmetry reasons. We note that only because of the substantially narrowed spectral lines (due to gain), both resonances are discernible. Without gain, both peaks merge because of the relatively broad width of the damped plasmonic resonances (cf. Figure 5b and Figure 5c). The marginal spectral shift from 513 nm for Δε_Dye_ = 0.004 (Figure 5d) to 511.3 nm in case of the lasing gain of Δε_Dye_ = 0.015 can be attributed to slight differences in the dielectric constant (Equation 1) of the PS sphere near the resonance for different gain levels, as well as to numerical discretization issues.

**Figure 7:**
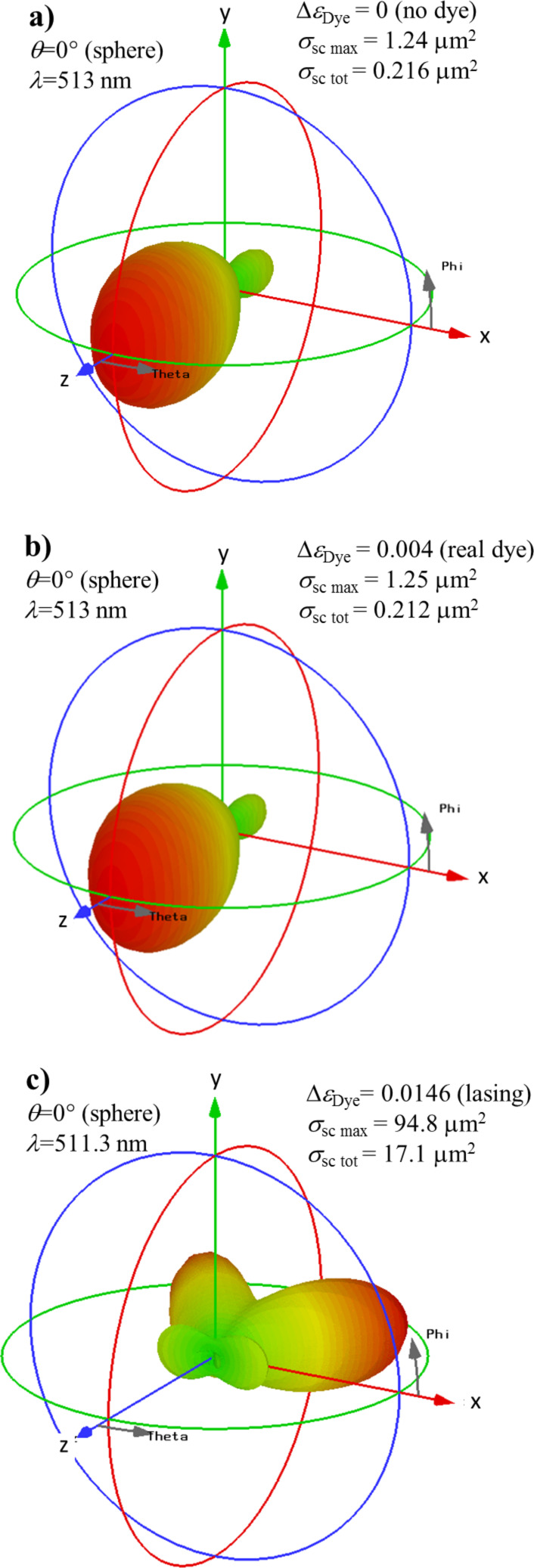
Differential scattering cross section σ_sc_, linear scale, for resonant semi-shells with thickness *h* = 16 nm at λ ≈ 513 nm. The irradiation is from the (0,0) direction; the strength of the emission (gain, A = 0, E = 1) is (a) Δε_Dye_ = 0 (no dye molecules at all), (b) 0.004 (commercially available dye concentration and total inversion) and (c) 0.0146 (lasing condition). The maximum of the differential and the total σ_sc_ are given in the legends.

In order to investigate the spasing modes in more detail, we plot the differential scattering cross sections in Figure 7 for the case of (0,0) illumination and for the cases of no dye molecules (Figure 7a), a currently commercially available concentration (Figure 7b) and for a dye doping close to the lasing threshold (Figure 7c). We plot the cross sections close to the resonances at 513 nm (Figure 7a and Figure 7b) and 511.3 nm (Figure 7c). While the differential scattering cross sections in case of undoped spheres (Figure 7a) and spheres doped with available concentrations (Figure 7b) look very similar and show Mie-type forward scattering, the pattern completely changes near the lasing threshold (Figure 7c). Here, the light is scattered predominantly in two lobes in the x–z plane, which point backward in z-direction (remember that the incident wave is along the positive z-direction). Further, the total scattering cross section is approx. 0.2 µm^2^ in case of no dye doping and commercially available doping, but increases significantly to approx. 17.1 µm^2^ close to the lasing threshold [29].

**Figure 8:**
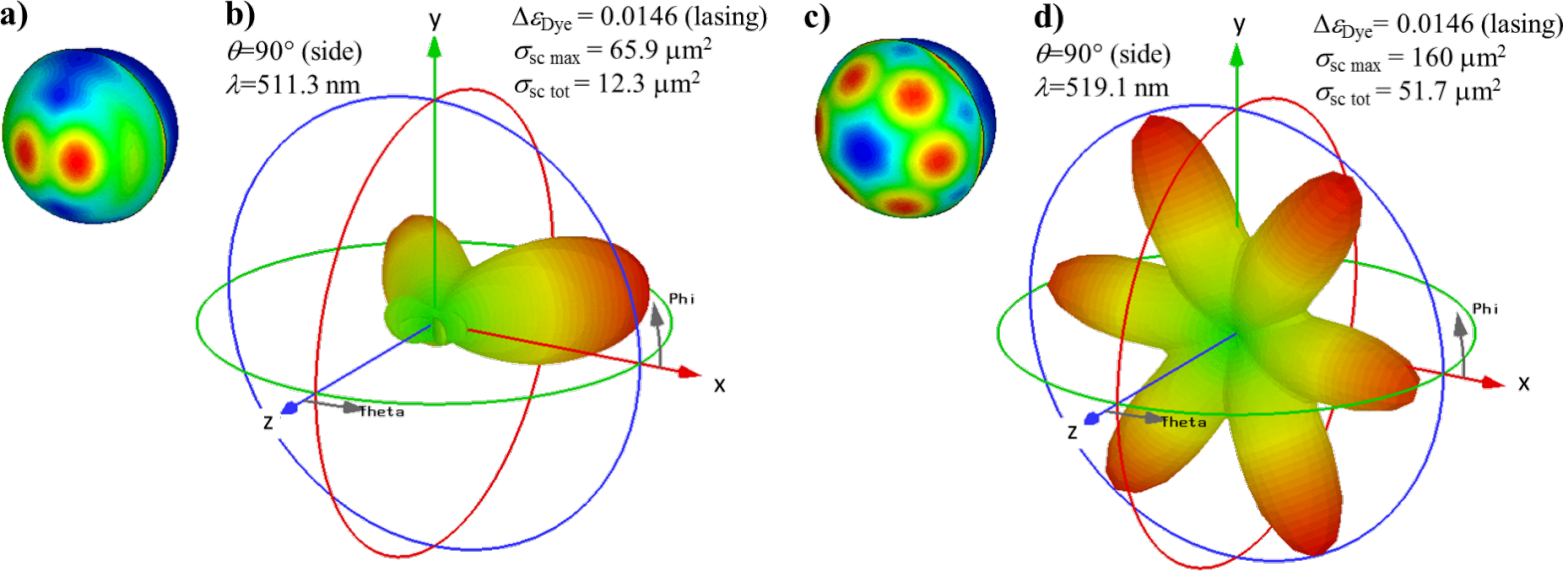
(a,c) Field enhancement |***E***|/*E*_0_ for two modes with gain close to the lasing threshold. The z-polarized incoming plane wave propagates in x-direction (θ = 90°, φ = 0°). The two wavelengths 511.3 nm (a) and 519.1 nm (c) are close to the two peaks in Figure 6d. The semi-shell thickness *h* = 16 nm, and a gain strength Δε_Dye_ = 0.0146 (near lasing condition) are assumed. (b,d) Differential scattering cross sections σ_sc_ , linear scale. The maxima of differential and total σ_sc_ are given in the legends.

Figure 8 shows the (near-)field enhancement |***E***|/*E*_0_ close to the semi-shell structure and the differential scattering cross sections in the case of (90,0) illumination, for a near-threshold gain (A = 0, E = 1) and for the two resonances (cf. Figure 6d, blue curves). Figure 8a and Figure 8b are calculated at 511.3 nm, close to the first spasing peak, and Figure 8c, and Figure 8d are calculated at 519.1 nm, close to the second peak that cannot be excited with (0,0) illumination. While the near-field distribution for the peak at 511.3 nm (Figure 8a) looks very similar (but with much stronger fields) to the case of the undoped semi-shell structure (Figure 3b), the near field distribution at 519.1 nm (Figure 8c) looks very different and resembles an (*l* = 3, *m* = 3) orbital. Clearly, this eigen-resonance is easily overlooked in experiments, in which semi-shells with insufficient or no gain are investigated because it would be hidden within the natural width of the *l* = 2 resonance at 513 nm, and it can also be overlooked in numerical calculations because the wavelength stepping needs to be small enough not to miss it.

The mode at 511.3 nm can be excited with both the (0,0) and the (90,0) illumination directions. Hence we can compare the scattering diagrams for both cases at a gain close to threshold (Figure 7c and Figure 8b, respectively). The total scattering cross section is larger in the case of the (0,0) illumination than for the (90,0) direction (17.1 µm^2^ versus 12.3 µm^2^, respectively), but the angular distributions look very similar. In stark contrast, the 519.1 nm mode, which is excitable only through (90,0) illumination, shows a decisively different far-field scattering distribution (Figure 8d). It is also worth mentioning that the near-field distributions (Figure 3b, Figure 8a, Figure 8c) look very different to the far-field differential cross sections (Figure 7c, Figure 8b, Figure 8d).

**Figure 9:**
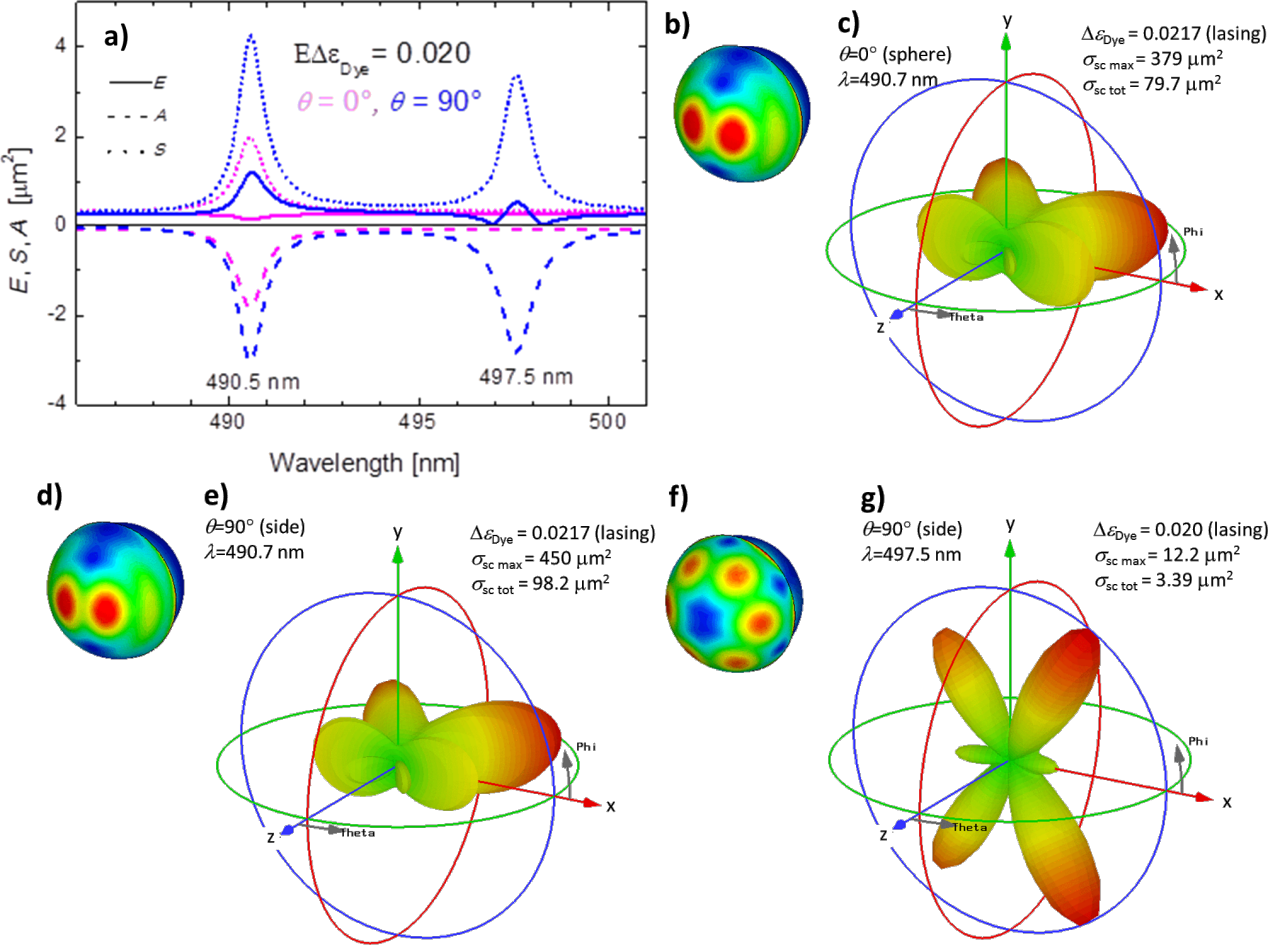
(a) Cross sections in case of a semi-shell of thickness *h* = 20 nm near the lasing threshold of Δε_Dye_ = 0.020, A = 0, E = 1, extinction (solid lines), scattering (dotted lines) and absorption cross sections (dashed lines) for illumination from the (0,0) direction (magenta lines) or (90,0) direction (blue lines). (b–g) Near-field distribution and differential scattering cross sections; (b,c): for the 491 nm resonance and the (0,0) illumination; (d,e): for the 491 nm resonance and the (90,0) illumination; and (f,g): for the 497.5 nm resonances and (90,0) direction. Note that in the case of (b–e) Δε_Dye_ = 0.0217 was used instead of 0.020 as in (a) and (f,g), see main text.

This observation becomes even more prominent when we look at a semi-shell of a slightly increased thickness of *h* = 20 nm instead of 16 nm. Figure 9a shows the extinction (solid lines), scattering (dotted lines) and absorption cross sections (dashed lines) for illumination from the (0,0) direction (magenta lines) and the (90,0) direction (blue lines) and hence Figure 9a directly corresponds to Figure 6d. Again, a shorter wavelength resonance, this time near 491 nm, is excitable for both illumination directions (0,0) and (90,0), while the longer wavelength “axial” resonance, this time at 497.5 nm, is only excitable in case of (90,0) illumination. Note, that appreciably larger gain of approximately Δε_Dye_ = 0.0217 is needed in case of the *h* = 20 nm Ag semi-shell to reach the lasing threshold as compared to only Δε_Dye_ = 0.0146 in case of the thinner shell of *h* = 16 nm. This is explained by two facts: a) larger losses in a thicker metal, and b) the absorption peak of the *h* = 16 nm shell coincides with the Lorentzian gain profile (cf. Figure 5c), while the peak of the *h* = 20 nm shell does not. Panels (b,c), (d,e), and (g,f) of Figure 9 show the near-field distributions and the far-field scattering for the semi-shell structure with *h* = 20 nm for the following conditions: (0,0) illumination at 490.7 nm (b,c), (90,0) illumination at 490.7 nm (d,e) and (90,0) illumination at 497.5 nm (f,g). Note that in case of (b–e) Δε_Dye_ = 0.0217 was used instead of 0.020 as in (a) and (f,g) because the lasing thresholds for the 497.5 nm resonance and the 490.7 nm resonance turned out to be slightly different. These graphs should be compared to the respective graphs for the *h* = 16 nm Ag semi-shells, namely Figure 7c for the (0,0) illumination and Figure 8 for the (90,0) illumination. We start with the discussion of the long-wavelength resonances 519.1 nm (Figure 8c,d) and 497.5 nm (Figure 9f,g), which can only be excited with the axially polarized (90,0) illumination. The near-field distributions (Figure 8c and Figure 9f) as well as the differential scattering cross sections (Figure 8d and Figure 9g) look very similar. Only the relative strengths of the six lobes show some differences. The same tendency is observed for the “transverse” shorter-wavelength resonances at 511.3 and 491 nm (compare Figures 7c, 8a,b and 9b–e). We would like to note that the patterns of the differential cross sections change dramatically within a narrow range of parameters, which can be easily overlooked in the experiments as well as in the simulations. The present scattering diagrams differ strongly from the more symmetric ones reported in [51], in which smaller and thinner semi-shell structures were simulated. (The results from this report are fully reproducible by our numerical framework). All this corroborates the observation that one cannot easily foretell the pattern of the differential scattering cross sections from the appearance of the plasmonic eigenmode.

**Figure 10:**
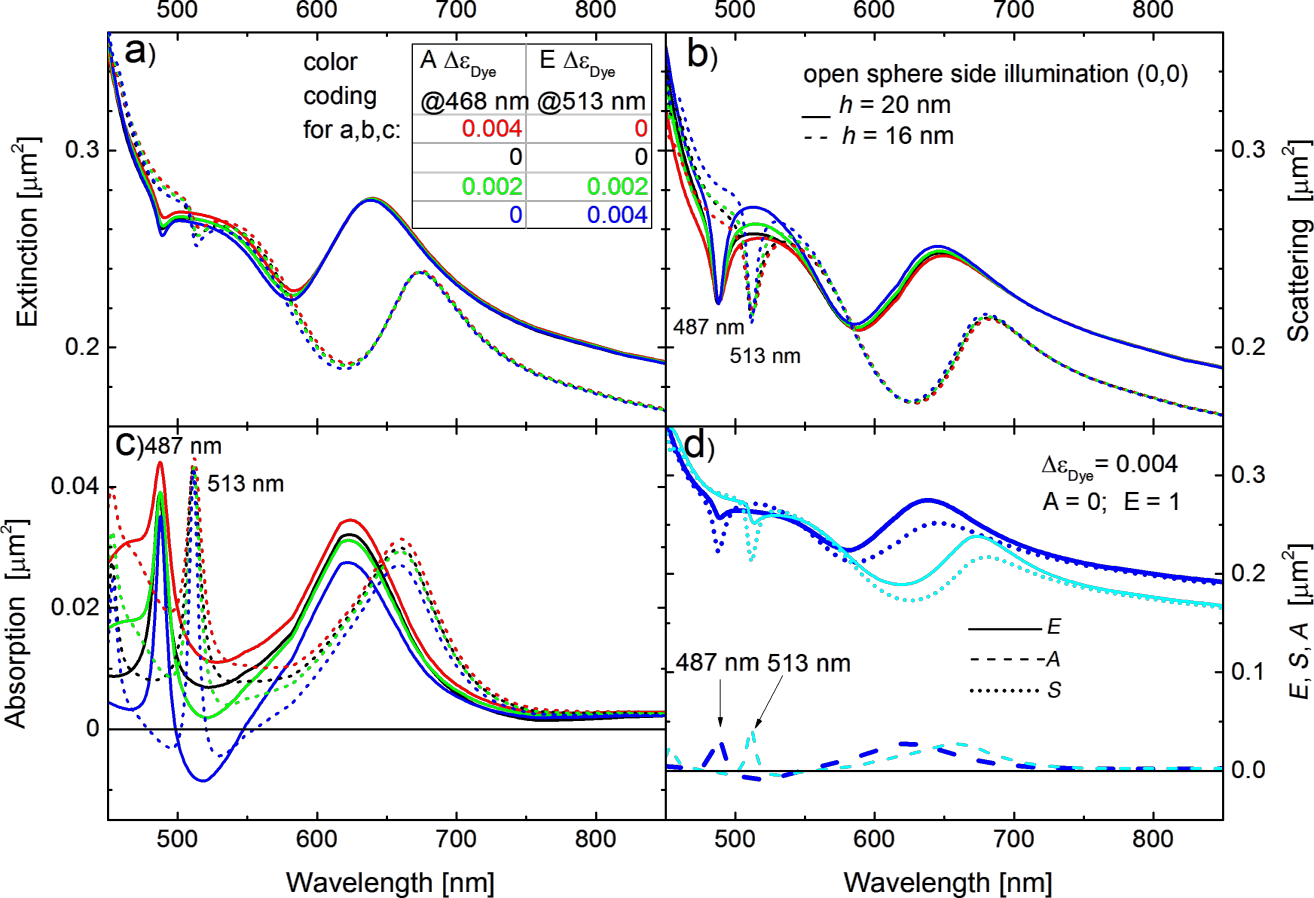
Differential cross sections for semi-shell thicknesses *h* = 20 nm (solid curves) and *h* = 16 nm (dashed curves), at dye concentrations as in commercially available PS spheres. All other parameters are the same as in Figure 2. For *h* = 20 nm the absorption becomes negative near 513 nm, while for *h* = 16 nm it becomes negative near 500 and 530 nm. The structures show overcompensation of absorption at these wavelengths but no lasing yet. (d) Comparison of all cross sections on one plot for Δε_Dye_ = 0.004, A = 0.004, E = 1 for both thicknesses *h* = 20 nm (dark blue), and *h* = 16 nm (cyan).

Lastly, let us discuss, which spectroscopic footprints can be expected from a commercially available dye-doping level that yields Δε_Dye_ = 0.004. Obviously, one cannot expect spasing, because a 3.7 times larger dye concentration is mandatory for that in the current geometry. However, already at moderate doping levels, some unusual spectroscopic signals should be observable. Figure 10 compares the differential cross sections for semi-shell thicknesses *h* = 20 nm (solid curves) and *h* = 16 nm (dashed curves), at dye concentrations that can be found in commercially available PS spheres. All other parameters are the same as in Figure 2. The most striking result is that for *h* = 20 nm the absorption becomes negative near 513 nm, and for *h* = 16 nm it becomes negative near 500 and 530 nm. This means that the structures show an overcompensation of absorption at these wavelengths but no lasing yet. Changes in absorption for such concentrations are actually more pronounced for the off-resonance thickness *h* = 20 nm. Further, the features in the scattering spectrum are sharpened by gain and so do the (positive) peaks in the absorption spectrum at wavelength ranges, at which the absorption is not yet overcompensated, for instance the peaks at 487 nm in the case of the *h* = 20 nm semi-shells (Figure 10 c, solid lines). Finally, Figure 10d compares all cross sections on one plot for the full inversion of the dye molecules with realistic concentration for both thicknesses *h* = 20 nm (dark blue), and *h* = 16 nm (cyan). In Figure 10d the solid curves refer to extinction (*E*), dotted to scattering (*S*) and dashed to absorption (*A*).

## Conclusion

Dye-doped polystyrene spheres capped with a thin semi-shell of silver show rich spectra of absorption, scattering and extinction, which contain many peaks and shoulders. Without gain, i.e., as long as the dye molecules are not pumped, the spectra are broad and hiding some of the eigenmodes. However, these become clearly observable when the gain is switched on, which leads to a compensation of absorption and hence sharpens the plasmon resonances. Specifically, we showed that a mode of *l* = 3, *m* = 3 symmetry is only 8 nm away from a *l* = 2 mode. This tiny difference can certainly not be resolved without gain-narrowing of plasmonic resonances. We deliberately focused on the discussion of the gain that is required for spectral sharpening and compared the required gain to the gain available in commercially available polystyrene spheres. We find that for the investigated geometries spasing requires gain levels less than 3.7 times higher than those in commercially available dye-doped spheres. Furthermore, the directionality of the differential scattering cross section changes with the amount of gain, depends on the fine details of the structure geometry and is sensitive to the direction of illumination. All this demands a high manufacturing accuracy of semi-shell based spasers.

With gain, some “conventional intuition” might become wrong. For example that lower multipolar modes are usually the stronger ones. This intuition is based on the expansion into powers of the Mie-parameter (*a*/λ) where *a* is the typical structure size. This expansion still holds. However, when the denominator in one of the scattering/absorption terms goes to zero, this term dominates, irrespectively of the power of (*a*/λ) associated with it. Some of the higher modes may dominate simply because they best match the spectral bandwidth of the gain media.

## Numerical

Here we summarize several subtleties, crucial for reliable simulations. Conventional finite difference time domain (FDTD) approaches require an analytical approximation of the dispersion of the metal dielectric constant [59], which is not always satisfactory. Active media with optical gain may introduce instabilities into FDTD codes, unless auxiliary differential equations with gain saturation are used [60]. We tackle these difficulties using the (mainly iterative) frequency domain solver of the CST MWS^®^ software with open boundaries, realized by perfectly matched layers (PML, 10 mesh layers). The computational domain (without PML) is a cube with a side of about 1 μm. For excitation, a linearly polarized plane wave is applied. No symmetries were used in order to make the numerical framework fully suitable for oblique incidence under angles (θ ≠ 0, φ ≠ 0) and for asymmetric structures.

Special care has to be taken of sharp edges and vertices leading to numerical hot spots, which are, however, unrealistic because in experiments all vertices will show some natural rounding and even if very large curvatures could be realized, ultra-high field enhancement is unphysical on a scale below 1 nm because of nonlocality and quantum effects [61–62]. We used blended edges with radii of curvature *R*_blend_ = 5 nm.

The numerically prepared Lorentzian gain can be read into the CST software package as a list of ε values only if ε″ is artificially made positive. Afterwards, the properties of the material can be manually reverted to ε″ < 0 in the “history list”. The CST software calculates scattering and absorption cross sections σ_sc_, σ_ab_ by using internal algorithms. However, the absorption calculation fails for the active structures, when σ_ab_ becomes negative. For this reason, a Power Flow Monitor through a spherical face surrounding the structure was created, and the integral power flow through it was recalculated into σ_ab_. The extinction cross section is always defined as σ_ex_ = σ_sc_ + σ_ab_, irrespectively of the sign of σ_ab_. Far-field patterns are calculated by the internal CST routines, which project the fields on the sides of the bounding box into the far-fields via fast Fourier transform.

Adaptive meshing is a must for the accurate representation of the fields in the regions where their gradients are high [63], though it affects less such global characteristics as σ_ex_, σ_sc_ and σ_ab_. For time-efficient calculations distributed computing (DC) and parallelization capabilities of the CST package were used, usually with six solver servers, each calculating its own frequency point independently in parallel, and threaded over 2 CPUs. In a typical run, 12 employed CPUs needed a wall-time of about 2 h for the full spectra with about 300 frequency points. Usually 10 adaptive mesh runs were sufficient, resulting in 80–90 kcells, with the cell edge length varying between ≈1 nm near (smoothened) sharp metallic features and ≈50 nm in the free air regions.

## References

[1] Kreibig U, Vollmer M, Toennies J P (1995). Optical Properties of Metal Clusters.

[2] Zhou H S, Honma I, Komiyama H, Haus J W (1994). Phys Rev B.

[3] Averitt R D, Sarkar D, Halas N J (1997). Phys Rev Lett.

[4] Takei H (1999). J Vac Sci Technol, B.

[5] Love J C, Gates B D, Wolfe D B, Paul K E, Whitesides G M (2002). Nano Lett.

[6] Liu J, Maaroof A I, Wieczorek L, Cortie M B (2005). Adv Mater.

[7] Coyle S, Netti M C, Baumberg J J, Ghanem M A, Birkin P R, Bartlett P N, Whittaker M (2001). Phys Rev Lett.

[8] Charnay C, Lee A, Man S-Q, Moran C E, Radloff C, Bradley R K, Halas N J (2003). J Phys Chem B.

[9] Lassiter J B, Knight M W, Mirin N A, Halas N J (2009). Nano Lett.

[10] Ye J, Van Dorpe P, Van Roy W, Lodewijks K, De Vlaminck I, Maes G, Borghs G (2009). J Phys Chem C.

[11] Cole R M, Baumberg J J, Garcia de Abajo F J, Mahajan S, Abdelsalam M, Bartlett P N (2007). Nano Lett.

[12] Cortie M, Ford M (2007). Nanotechnology.

[13] Wang Q, Tang C, Chen J, Zhan P, Wang Z (2011). Opt Express.

[14] Lacharmoise P D, Tognalli N G, Goñi A R, Alonso M I, Fainstein A, Cole R M, Baumberg J J, Garcia de Abajo J, Bartlett P N (2008). Phys Rev B.

[15] Zhang Y, Barhoumi A, Lassiter J B, Halas N J (2011). Nano Lett.

[16] King N S, Li Y, Ayala-Orozco C, Brannan T, Nordlander P, Halas N J (2011). ACS Nano.

[17] Ding B, Hrelescu C, Arnold N, Isic G, Klar T A (2013). Nano Lett.

[18] Himmelhaus M, Takei H (2000). Sens Actuators, B.

[19] Lal N N, Soares B F, Sinha J K, Huang F, Mahajan S, Bartlett P N, Greenham N C, Baumberg J J (2011). Opt Express.

[20] Dunbar R B, Pfadler T, Lal N N, Baumberg J J, Schmidt-Mende L (2012). Nanotechnology.

[21] Mahajan S, Richardson J, Brown T, Bartlett P N (2008). J Am Chem Soc.

[22] Mahajan S, Cole R M, Soares B F, Pelfrey S H, Russell A E, Baumberg J J, Bartlett P N (2009). J Phys Chem C.

[23] Steuwe C, Kaminski C F, Baumberg J J, Mahajan S (2011). Nano Lett.

[24] Sudarkin A N, Demkovich P A (1989). Sov Phys - Tech Phys.

[25] Klar T A, Kildishev A V, Drachev V P, Shalaev V M (2006). IEEE J Sel Top Quantum Electron.

[26] Xiao S, Drachev V P, Kildishev A V, Ni X, Chettiar U K, Yuan H-K, Shalaev V M (2010). Nature.

[27] Campione S, Capolino F (2012). Nanotechnology.

[28] Campione S, Albani M, Capolino F (2011). Opt Mater Express.

[29] Noginov M A, Zhu G, Bahoura M, Adegoke J, Small C E, Ritzo B A, Drachev V P, Shalaev V M (2006). Opt Lett.

[30] Strangi G, De Luca A, Ravaine S, Ferrie M, Bartolino R (2011). Appl Phys Lett.

[31] Bergman D J, Stockman M I (2003). Phys Rev Lett.

[32] Lawandy N M (2004). Appl Phys Lett.

[33] Protsenko I E, Uskov A V, Zaimidoroga O A, Samoilov V N, O'Reilly E P (2005). Phys Rev A.

[34] Gordon J A, Ziolkowski R W (2007). Opt Express.

[35] Zheludev N I, Prosvirnin S L, Papasimakis N, Fedotov V A (2008). Nat Photonics.

[36] Stockman M I (2010). J Opt (Bristol, U K).

[37] Stockman M I (2007). Phys Rev Lett.

[38] Mackay T G, Lakhtakia A (2007). Phys Rev Lett.

[39] Kinsler P, McCall M W (2008). Phys Rev Lett.

[40] Wuestner S, Pusch A, Tsakmakidis K L, Hamm J M, Hess O (2010). Phys Rev Lett.

[41] Stockman M I (2011). Phys Rev Lett.

[42] Hill M T, Oei Y-S, Smalbrugge B, Zhu Y, de Vries T, van Veldhoven P J, van Otten F W M, Eijkemans T J, Turkiewicz J P, de Waardt H (2007). Nat Photonics.

[43] Nezhad M P, Simic A, Bondarenko O, Slutsky B, Mizrahi A, Feng L, Lomakin V, Fainman Y (2010). Nat Photonics.

[44] Lee J H, Khajavikhan M, Simic A, Gu Q, Bondarenko O, Slutsky B, Nezhad M P, Fainman Y (2011). Opt Express.

[45] Ding K, Liu Z C, Yin L J, Hill M T, Marell M J H, van Veldhoven P J, Nöetzel R, Ning C Z (2012). Phys Rev B.

[46] Kwon S-H, Kang J-H, Seassal C, Kim S-K, Regreny P, Lee Y-H, Lieber C M, Park H-G (2010). Nano Lett.

[47] Oulton R F, Sorger V J, Zentgraf T, Ma R-M, Gladden C, Dai L, Bartal G, Zhang X (2009). Nature.

[48] Lu Y-J, Kim J, Chen H-Y, Wu C, Dabidian N, Sanders C E, Wang C-Y, Lu M-Y, Li B-H, Qiu X (2012). Science.

[49] Noginov M A, Zhu G, Belgrave A M, Bakker R, Shalaev V M, Narimanov E E, Stout S, Herz E, Suteewong T, Wiesner U (2009). Nature.

[50] Pan J, Chen Z, Chen J, Zhan P, Tang C J, Wang Z L (2012). Opt Lett.

[51] Meng X, Guler U, Kildishev A V, Fujita K, Tanaka K, Shalaev V M (2013). Sci Rep.

[52] Tribelsky M I, Luk'yanchuk B S (2006). Phys Rev Lett.

[53] Fan X, Shen Z, Luk'yanchuk B (2010). Opt Express.

[54] Johnson P B, Christy R W (1972). Phys Rev B.

[55] Strickler S J, Berg R A (1962). J Chem Phys.

[56] Khurgin J B, Sun G (2012). Opt Express.

[57] Prodan E, Radloff C, Halas N J, Nordlander P (2003). Science.

[58] Baranov D G, Andrianov E S, Vinogradov A P, Lisyansky A A (2013). Opt Express.

[59] Vial A, Laroche T (2008). Appl Phys B.

[60] Prokopeva L J, Trieschmann J, Klar T A, Kildishev A V (2011). Proc SPIE.

[61] Garcia de Abajo F J (2008). J Phys Chem C.

[62] Zuloaga J, Prodan E, Nordlander P (2009). Nano Lett.

[63] Hoffmann J, Hafner C, Leidenberger P, Hesselbarth J, Burger S (2009). Proc SPIE.

